# Same same but different: the case of olfactory imagery

**DOI:** 10.3389/fpsyg.2014.00034

**Published:** 2014-02-03

**Authors:** Artin Arshamian, Maria Larsson

**Affiliations:** Gösta Ekman Laboratory, Department of Psychology, Stockholm UniversityStockholm, Sweden

**Keywords:** imagery, olfaction, consciousness, comparative, expertise

## Abstract

In the present work we present an overview of experimental findings corroborating olfactory imagery observations with the visual and auditory modalities. Overall, the results indicate that imagery of olfactory information share many features with those observed in the primary senses although some major differences are evident. One such difference pertains to the considerable individual differences observed, with the majority being unable to reproduce olfactory information in their mind. Here, we highlight factors that are positively related to an olfactory imagery capacity, such as semantic knowledge, perceptual experience, and olfactory interest that may serve as potential moderators of the large individual variation.

## INTRODUCTION

“We lay no great weight upon these results, though they are evidently in accord with those obtained with vision and audition” (Perky, 1910, p. 441). This statement summarized the opinion stated about the nature of olfactory imagery in one of the first studies targeting mental imagery. However, more than a century later the scientific evidence pertaining to our ability to form olfactory images is yet scarce although the topic has received an upsurge of interest during the past years. The aim of this work is to summarize the current findings from three angles; similarity, difference, and plasticity. First, we show that olfactory imagery shares many of the features known for visual and auditory imagery. Second, we propose that olfactory imagery is radically different in one important aspect; the large individual variation in the capacity to form olfactory images. Finally, we discuss factors that moderate the individual differences, such as semantic knowledge, perceptual experience, and olfactory interest.

## SIMILARITIES AMONG VISUAL, AUDITORY, AND OLFACTORY IMAGERY

Although some researchers have declared that we are unable to form olfactory images (Engen, 1982, 1991; Crowder and Schab, 1995; Herz, 2000), support for an olfactory imagery capacity is currently pervasive. The bulk of this work suggests that many features of the olfactory image are shared by visual and auditory imagery. **Table 1** provides an overview of some of these features based on experimental observations across the olfactory, visual, and auditory modalities. For example, multidimensional scaling studies have demonstrated a correspondence between visual perception and imagery in judgments of shapes (e.g., Shepard and Chipman, 1970) and within audition, a strong association between perceived and imagined musical timbre has been documented (e.g., Intons-Peterson et al., 1992). In the olfactory domain, correspondences for pleasantness, intensity, and familiarity ratings have been established between olfactory perception and imagery (e.g., Carrasco and Ridout, 1993; Sugiyama et al., 2006). Moreover, studies have demonstrated that both visual (e.g., Craver-Lemley and Reeves, 1992) and auditory (e.g., Segal and Fusella, 1970) imagery can interfere with perceptual thresholds for the same imagery modality. Likewise, Djordjevic et al. (2004) observed that participants that were asked to imagine an odor and later presented with either the same or different odor were less able to detect the latter. A finding that proved modality-specific. In patient studies, clinical manifestations of visual, auditory, and olfactory hallucinations have been observed for a range of conditions, such as epilepsy (visual: Panayiotopoulos, 1999; auditory: Korsnes et al., 2010; olfactory: West and Doty, 1995), and as a result of cocaine abuse (Siegel, 1978). Dream studies have demonstrated sensory specific components included in visual (e.g., MacCarely and Hoffman, 1981), auditory (e.g., Zadra et al., 1998), and olfactory dream reports (e.g., Stevenson and Case, 2005a). Ocular motor activity in visual imagery (e.g., Laeng and Teodorescu, 2002), and subvocalization in auditory imagery (Aleman and Wout, 2004) have been demonstrated as important factors during imagery. Similarly, the peripheral motor act of sniffing have been shown to influence mental imagery in a modality specific manner, as a blocking of the nostrils decrease olfactory (Bensafi et al., 2003) but not visual imagery (Arshamian et al., 2008). Observations promoting the olfactory image as one of our imagery modalities can also be derived from brain research. For example, research indicates that stimulation of sensory specific brain areas can induce modality specific imagery in vision (Diederich and Goetz, 2000), audition (Moriarity et al., 2001) and in olfaction by electrical stimulation of the olfactory bulb and tract (Kumar et al., 2012).

**Table 1:** Experimental observations corroborating olfactory imagery observations with the visual and auditory modalities.

Observations	Visual imagery	Auditory imagery	Olfactory imagery
**(I) Preserved properties between perception and imagery**
Correspondence in multidimensional scaling between perception and imagery. Psychophysical correspondence between perception and imagery.	Similar ratings of presented and imagined states of the US Shepard and Chipman (1970). Preserved time properties following mental rotation of three-dimensional objects Shepard and Metzler (1971).	Similar ratings of perceived and imagined musical timbre Halpern et al. (2004). Preserved pitch distance in auditory imagery Intons-Peterson et al. (1992).	Similar ratings between perceived and imagined odors Carrasco and Ridout (1993), Sugiyama et al. (2006). Preserved intensity for perceived and imagined odors Algom and Cain (1991).
**(II) Interference in perceptual thresholds as a function of imagery**	Visual imagery interference in vision Craver-Lemley and Reeves (1992).	Interference of imaged sounds on detection of auditory signals Segal and Fusella (1970).	Interference effects of odor imagery on odor detection Djordjevic et al. (2004).
**(III) Sensory specific hallucinations**
Hallucinations in schizophrenia.	Bracha et al. (1989).	George and Neufeld (1985).	Stedman and Clair (1998).
Hallucinations in patients with epilepsy.	Visual hallucinations Panayiotopoulos (1999).	Auditory hallucinations Korsnes et al. (2010).	Olfactory hallucinations West and Doty (1995).
Hallucinations associated with migraine.	Visual hallucinations Schott (2007).	Auditory hallucinations Rubin et al. (2002).	Olfactory hallucinations Fuller and Guiloff (1987).
Hallucinations associated with drug abuse.	Cocaine induced visual hallucinations Siegel (1978).	Cocaine induced auditory hallucinations Siegel (1978).	Cocaine induced auditory hallucinations Siegel (1978).
	Visual hallucinations during alcohol withdrawal Bayard et al. (2004).	Auditory hallucinations during alcohol withdrawal Saravay and Pardes (1967).	Olfactory hallucinations during alcohol withdrawal Stevenson and Langdon (2012).
**(IV) Dream reports containing a sensory component**	Visual dreams MacCarely and Hoffman (1981).	Auditory dreams Zadra et al. (1998).	Olfactory dreams Stevenson and Case (2005a).
**(V) Volitional mental imagery**
Reports of volitional imagery involving a sensory component. Correlations between different modalities in vividness for volitional imagery. Female advantage in reported volitional imagery vividness.	Involving vividness ratings of imagined pictures Marks (1973). Correlation between visual and auditory imagery Hubbard (2010). More vivid auditory White et al. (1977).	Involving vividness ratings of imagined sounds Willander and Baraldi (2010). Correlation between visual and auditory imagery Hubbard (2010). More vivid auditory White et al. (1977).	Involving vividness ratings of imagined smells Gilbert et al. (1998). Correlation between olfactory and visual imagery Stevenson and Case (2005a). More vivid visual images White et al. (1977).
**(VI) Peripheral motor activity**
Correspondence between sensory specific peripheral motor activity and imagery. Diminished mental imagery following interference of peripheral motor activity.	Similarity between scanpaths made when viewing objects and when later imagining the same object Brandt and Stark (1997). Decrease in visual imagery when interfering with the ocular motor activity Laeng and Teodorescu (2002).	Subvocalization during auditory imagery for verbal materials and familiar melodies Hubbard (2010). Decrease in auditory imagery when subvocalization is blocked Aleman and Wout (2004).	Similarity between olfacto-motor activity during imagery and perception of the same odor Bensafi et al. (2003). Decrease in olfactory imagery when sniffing is blocked, e.g., Bensafi et al. (2003), Arshamian et al. (2008).
**(VII) Activation of sensory specific brain areas during imagery**	Activation of primary and secondary visual cortices, Ganis et al. (2004).	Activation of primary and secondary auditory cortices, Halpern et al. (2004).	Activation of primary and secondary olfactory cortices, e.g., Djordjevic et al. (2005), Bensafi et al. (2007).
**(VIII) Reports of mental sensation following electrical stimulation of the brain**	Visual imagery following stimulation of the nucleus subthalamicus Diederich and Goetz (2000).	Auditory imagery following stimulation of the lateral temporal cortex Moriarity et al. (2001).	Olfactory imagery following stimulation of olfactory bulb and tract Kumar et al. (2012).
**(IX) The effect of expertise on mental imagery**
The effect of domain specific expertise knowledge on mental imagery.	Enhanced recall of rapidly and randomly presented chess positions in professional chess players Gobet and Simon (1996).	Increased pith and temporal acuity in auditory imagery as a function of musical training Janata and Paroo (2006).	Olfactory experts reportingmore vivid olfactory images than nonexperts, e.g., Gilbert et al. (1998).
**(X) The effect of expertise on modality specific brain plasticity**
Functional reorganization during mental imagery.	Functional reorganization for expert mnemonists for visual objects Maguire et al. (2002).	Functional reorganization as a function of musical expertise Groussard et al. (2010).	Experience induced functional reorganization in perfumers Plailly et al. (2012).
Structural reorganization	Structural reorganization among expert GO players Lee et al. (2010).	Effects of musical expertise on structural plasticity Groussard et al. (2010).	Experience induced structural reorganization in perfumers Delon-Martin et al. (2013).

## INDIVIDUAL DIFFERENCES IN OLFACTORY IMAGERY

Although similar in many respects, the capacity to form olfactory images differs from that observed in visual and auditory imagery. For instance, only a minimal portion of the population is unable to create visual images (Kosslyn et al., 2006), whereas the olfactory modality is documented as the sense with the fewest instances of volitional imagery and with the highest frequency of individuals reporting that mental imagery never has occurred (Stevenson and Case, 2005b). Also, if an odor image is successfully produced it is typically experienced as less vivid than images generated from other modalities (Betts, 1909; Sheehan, 1967; White et al., 1978; Ashton and White, 1980). Also, Olivetti Belardinelli et al. (2009) demonstrated that self-rated reports of olfactory imagery vividness, unlike for example vividness ratings in visual or tactile imagery, did not correlate with modality specific brain activation. In an evolutionary context it is highly likely that the selection pressure for an imagery capacity was stronger for the visual and auditory systems among the early hominoids than for most other mammals. However, this circumstance does not entail that the capacity to form olfactory images reached extinction. A weaker selection pressure more likely resulted in a larger individual variation in the capacity to evoke olfactory images. Hence, the less vibrant olfactory image may be a direct result from an environment favoring proficient imagery abilities in the visual and auditory modalities. In this vein, it is of interest to note that Lawless (1997) reported that the frequencies of olfactory imagery (ranging from never to often) and the image vividness (ranging from 0 to 100%) were more normally distributed than visual and auditory imagery. For example, whereas all study participants had experienced a visual image a significant proportion reported never experiencing olfactory images. Also, the experienced vividness for visual and auditory images were heavily shifted towards vividness ratings over 75%, while more than half of the reported olfactory images had a vividness rating of 25% or less. Hence, an olfactory imagery capacity was probably of little survival value for the anatomically modern human. However, in animals, such as rats, where the olfactory sense is a main percept for survival, the capacity to form olfactory images appears exceptional. For example, April et al. (2013) demonstrated that rodent working memory capacity, as measured by odor span task, was in the magnitude of 72 stimuli, and that its structure more resembled an episodic-like memory. It has been hypothesized that the evolution of working memory, and thus imagery capacity, was partially evolved in the context of planning, recalling, and reasoning appropriately about food caching (see Carruthers, 2013 for a review). Thus, in contrast to rats the ability to evoke olfactory images probable had little, if any use for the modern humans with an evolved visual and auditory imagery capacity. However, as noted below, olfactory imagery may still play an important role in the everyday life.

## FACTORS MODERATING OLFACTORY IMAGERY CAPACITY

Most of the arguments raised for the inability to experience smells without external stimuli gain support from studies targeting differences found between olfaction and other sensory modalities. One example speaking to this view is that evidence is yet inconclusive regarding the nature of olfactory working memory in humans (Engen, 1991; Wilson and Stevenson, 2006; Zelano et al., 2009). Other concerns pertain to the well-documented difficulty to name odors, while the corresponding objects to these odors are easy to name when seen (Cain, 1979; Larsson et al., 1999; Olofsson et al., 2013). As a functional working memory capacity and semantic knowledge are considered as prerequisites for an imagery capacity in general, these two factors appear as fundamental for the integrity of olfactory imagery. Hence, activities that may promote the development of these factors, such as perceptual practice and odor-name learning, may contribute positively to the individual variation.

Stevenson et al. (2007) examined the relationship between odor identification and the ability to form odor images. The results showed that odors that were difficult to name also were difficult to imagine and that prior learning of the odor names exerted a positive effect on imagery capacity. Moreover, Tomiczek and Stevenson (2009) reported that odor imagery priming was prevalent only among good odor namers and appeared to be the result of a generic activation of olfactory neural networks when the participants tried to form an odor image. Importantly, Tomiczek and Stevenson (2009) suggested that this could occur in dependently of any consciously reported olfactory image. Thus, the act of trying to imagine an odor could result in a behavioral change that is not accompanied by a consciousness experience of that odor (Stevenson, 2009).

Other factors that have been linked to olfactory imagery are olfactory dreams and interest. For example, Stevenson and Case (2005a) explored factors such as odor interest, prevalence of odor dreams, and self-rated olfactory imagery in relation to olfactory performance. The results revealed that individuals who experienced olfactory dream content identified more odors correctly than non-olfactory dreamers. Concomitantly, prevalence of olfactory dreams was positively related to olfactory imagery capacity and a higher interest of odors in general. Moreover, Arshamian et al. (2011) selected individuals with either high or low olfactory awareness as indexed by rated imagery ability, prevalence of olfactory dreams, and odor interest. The results replicated and extended Stevenson and Case (2005a) by showing that high olfactory awareness not only was related to a more proficient spontaneous odor identification but also to a better retention of olfactory information as compared to the group with low awareness. Notably, the better episodic memory performance was not driven by a higher proficiency to verbalize information (Larsson, 1997; Larsson and Bäckman, 1997). Hence it is possible that persons experiencing olfactory dreams and have high olfactory interest may be less dependent on semantic processes when remembering odors. Moreover, the individual variation in interest may partially be attributed to differences in attraction and attention towards odors. For example, Bensafi and Rouby (2007) showed that individuals who scored high in olfactory imagery also had a higher ability to experience pleasure, and perceived pleasant odors as more pleasant and familiar than poor olfactory imagers.

## PLASTICITY IN OLFACTORY IMAGERY CAPACITY AMONG NOVICES AND EXPERTS

Studies indicate that indirect and moderate opportunities to stimulate olfactory imagery through perceptual exposure are effective. Recently, Bensafi et al. (2013) compared olfactory and auditory imagery in individuals that cooked on a daily basis with a group that played music and was musically trained with a group of control participants who neither cooked nor played any instruments. The results showed that individuals that cooked had shorter response times than musical and controls in judgments associated with olfactory imagery, but not auditory imagery, whereas response times in auditory imagery were shorter for the musical group. Hence, this observation suggests that indirect and moderate perceptual practice may exert positive effects on modality specific behavior.

Research focusing on training the sense of smell has mainly focused on wine experts and perfumers (e.g., Lawless, 1984; Melcher and Schooler, 1996; Parr et al., 2002; Plailly et al., 2012). One observation is that olfactory experts, such as perfumers, exhibit a higher volitional olfactory imagery capacity than novices (Gilbert et al., 1998) and that the skills primarily result from a higher conceptual knowledge, rather than an inherent higher chemosensory sensitivity (De Beni et al., 2007). For example, Melcher and Schooler (1996) reported that wine experts compared to novices performed better in a “triangle test” where one target wine had to be picked out from a group of three. Experts and novices had to verbally describe the target wine before picking it out after a 4-min retention interval. Whereas verbalization did not affect wine experts in recognition, the novices showed impaired wine recognition. Similarly, it has been shown that wine experts are less susceptible to verbal overshadowing than novices (Parr et al., 2002). Several studies report that the superior performance of wine experts is largely determined by their ability to form appropriate verbal descriptors that focuses on the sensory quality (Lawless, 1984). In this vein, Engen and Ross (1973) reported that odor memory decreased if participants gave loosely related verbal labels to the odors compared to odors that were not labeled. In line with this idea, Fiore et al. (2012) tested if short-term memory for flavors could be influenced by olfactory imagery and the usage of appropriate verbal labels in amateurs. The results showed that imagination of a wine flavor with descriptive oenological adjectives, enhanced memory for the specific wine. In contrast, Parr et al. (2002) observed that wine experts performed better in odor recognition memory, although there were no group differences in odor identification and verbal memory. Hence, verbal codes were not necessary for a better recognition among experts suggesting the use of other strategies (cf. Arshamian et al., 2011).

However, not only conceptual odor knowledge shows positive benefits from training. Plailly et al. (2012) used functional magnetic resonance imaging (fMRI) to study changes in functional activity as a function of extensive olfactory training. Student and professional perfumers were presented with odor names and were asked to create an olfactory image for each odor name. In general, the anterior part of the piriform cortex appeared as a crucial area for olfactory imagery, although students showed more activation in the posterior part of the piriform cortex. This indicated that that the two groups used different strategies when generating odor images. Interestingly, the duration of work experience in perfumers also modified the neural activity. A longer work experience was related to less brain activity in areas associated with olfactory imagery and perception (i.e., piriform cortex, orbitofrontal cortex, and the hippocampus). This type of experience-induced decrease in functional brain activity has been reported for other modalities, such as vision (Maguire et al., 2002) and audition (Ohnishi et al., 2001). However, caution should be made when drawing conclusions from olfactory cortex activity alone as several other factors, such as sniffing (Sobel et al., 1998), semantic labels denoting odors (González et al., 2006), cross-modal reactivation (Gottfried et al., 2002, 2004), and attention towards odors (Zelano et al., 2005, 2011) may activate olfactory cortex. Hence, activity in olfactory cortex may be conceived as a necessary, but not a sufficient condition for the integrity of olfactory imagery (see Royet et al., 2013, for a review). Plailly et al. (2012) also reported that the inferior temporal gyrus, an area involved in semantic memory processing (Irish et al., 2012), decreased its activity with increasing expertise. This observation may reflect that generation of an olfactory image is subserved by semantic memory, but that with more extensive olfactory knowledge the retrieval gets less dependent on semantic feedback. A follow-up study also demonstrated that the structural brain images were modified with olfactory expertise”(Delon-Martin et al., 2013). Specifically, perfumers had larger gray-matter volumes in areas associated with olfactory processing, which included the bilateral gyrus rectus/medial orbital gyrus and the anterior cingulate. Further, the gray-matter volume increased with experience in the primary olfactory cortex and in the left rectus/medial orbital gyrus. No differences in areas involved in semantic processing were reported suggesting that structural changes following extensive perceptual experience, and to some extent olfactory imagery training, were restricted to modality-specific areas, such as primary and secondary olfactory cortices.

## ODOR IMAGERY IN PERSONS WITH SMELL LOSS

Flohr et al. (Submitted) investigated the relationship between olfactory loss and the capacity to form olfactory images. Patients with olfactory loss and a control group with a normal sense of smell performed odor imagery tasks in the fMRI whilst also factors that could potentially activate olfactory cortex (e.g., sniffing) were controlled for. The study took advantage of results from studies indicating that odor imagery mimics that of olfactory perception. Specifically, both unpleasant odors and their mental images induce stronger activity in the piriform cortex and insula as compared to activity related to pleasant odors and their respective images (Bensafi et al., 2007). The results from Flohr et al. (submitted) showed that although patients with olfactory loss showed activity in areas associated with olfactory imagery, it was, unlike the control group, not related to the hedonic quality to-be-imagined. Also, the longer the duration of the smell loss the more activity in regions associated with olfactory imagery was observed. Thus, olfactory loss shows a reverse activation pattern than that observed among perfumers, which showed less activity with increasing experience (Plailly et al., 2012). The conclusion was that patients with olfactory loss were unable to evoke olfactory images similar to controls and that a regular exposure to olfactory information is crucial for successful imagery and that there may be a gradual memory loss of olfactory representations over time.

## CONCLUDING REMARKS

The capacity to form olfactory images in the normal population should be regarded as a continuous factor. At the opposite ends, individuals with anosmia and olfactory experts are located. Severe olfactory impairment and anosmia are associated with reductions in accessing conscious odor information whereas olfactory expertise is linked to a fluent and conscious retrieval of olfactory information (Flohr et al., submitted; Plailly et al., 2012). The majority of the population is, however, located at an intermediate position, where difficulties in experiencing and recreating an odor into a conscious image are typical. However, a continuous perceptual stimulation and exposure to olfactory information may eventually increase the likelihood to be able to recreate conscious olfactory percepts in the mind.

In conclusion, this overview suggests that the olfactory image shares many features with visual and auditory imagery although some major differences are evident. The most prominent discrepancy concerns the large individual differences reported for our capacity to reproduce a smell with our inner nose. Here, factors such as the identity of the odor, odor interest, and perceptual experience were discussed as potential moderators of the individual variation.

## AUTHOR CONTRIBUTIONS

Artin Arshamian and Maria Larsson jointly wrote the manuscript.

## Conflict of Interest Statement

The authors declare that the research was conducted in the absence of any commercial or financial relationships that could be construed as a potential conflict of interest.
